# Use of the gamma3™ nail in a teaching hospital for trochanteric fractures: mechanical complications, functional outcomes, and quality of life

**DOI:** 10.1186/1756-0500-5-651

**Published:** 2012-11-23

**Authors:** Benjamin Buecking, Christopher Bliemel, Johannes Struewer, Daphne Eschbach, Steffen Ruchholtz, Thorben Müller

**Affiliations:** 1 Department of Trauma, Hand, and Reconstructive Surgery, University of Giessen and Marburg GmbH, Location Marburg, Baldingerstrasse, Marburg, 35043, Germany

**Keywords:** Trochanteric fractures, Surgical education, Gamma3 nail, Outcome, Quality of life, Complications, Mortality

## Abstract

**Background:**

Trochanteric fractures are common fractures in the elderly. Due to characteristic demographic changes, the incidence of these injuries is rapidly increasing. Treatment of these fractures is associated with high rates of complications. In addition, the long-term results remain poor, with high morbidity, declines in function, and high mortality. Therefore, in this study, complication rates and patients’ outcomes were evaluated after fixation of geriatric trochanteric fractures using the Gamma3™ nail.

**Methods:**

Patients aged 60 years old or older, with pertrochanteric and subtrochanteric femoral fractures, were included. Patients with polytrauma or pathological fractures were excluded. Age, sex, and fracture type were collected on admission. In addition, data were recorded concerning the surgeon (resident vs. consultant), time of operation, and local or systemic perioperative complications. Complications were also collected at the 6- and 12-month follow-ups after trauma. Barthel Index, IADL, and EQ-5D measurements were evaluated retrospectively on admission, as well as at discharge and during the follow-up.

**Results:**

Ninety patients were prospectively included between April 2009 and September 2010. The patients’ average age was 81 years old, and their average ASA score was 3. The incision/suture time was 53 min (95% CI 46–60 min). Hospital mortality was 4%, and overall mortality was 22% at the 12-month follow-up. Eight local complications occurred (4 haematomas, 1 deep infection, 1 cutting out, 1 irritation of the iliotibial tract, 1 periosteosynthetic fracture). The incidence of relevant systemic complications was 6%. Forty-two percent of the patients were operated on by residents in training, without significant differences in duration of surgery, complication rate, or mortality rate. The Barthel Index (82 to 71, *p* < .001), IADL (4.5 to 4.3, *p* = .0195) and EQ-5-D (0.75 to 0.66, *p* = .068) values did not reach pre-fracture levels during the follow-up period of 12 months.

**Conclusion:**

The results showed a relatively low complication rate using the Gamma3™ nail, even if the nailing was performed by residents in training. The high mortality, declines in function, and low quality of life could probably be attributed to pre-existing conditions, such as physical status.

In summary, the Gamma3™ nail seems to be a useful implant for the nailing of trochanteric fractures, although further studies are necessary comparing different currently available devices.

## Background

Proximal femoral fractures are common fragility fractures in the elderly. These injuries have been identified as among the most serious health care problems affecting the elderly [1]. The estimated costs of these fractures are €2 billion to €4 billion per year in Germany [2]. Worldwide, the incidence of fractures of the proximal femur is increasing because of a demographic transition resulting in higher life expectancy [1,3-5]. For example, by 2050, 48% of the German population will be older than 65 years old, according to estimates from the Federal Statistics Office of Germany [6]. Trochanteric and subtrochanteric fractures amount to approximately half of proximal femoral fractures [7]. These fractures will occur more often in the future. Thus, the successful treatment of these fractures is becoming increasingly important.

In the infancy of the surgical treatment of trochanteric fractures, rigid combinations of femoral nails with lateral plates were used. High rates of cutting out, metal failure, or secondary fracture displacement were reported. Subsequently, dynamic fixation was introduced with the Ender nail or with the compression hip screw [8]. The sliding hip screw became the standard of care in these fractures for many years, with good results in minimally displaced and stable fractures. However, this osteosynthesis has not proved successful in complicated trochanteric fractures [9]. It is for this reason that intramedullary implants have been increasingly used as an alternative for these fracture types.

The first version of this intramedullary device was the Gamma™ nail. It was the first device that could be implanted minimally invasively, and it promised certain biomechanical advantages.

Initial randomised, controlled trials in the early 1990s, comparing the sliding hip screw with the Gamma™ nail, showed high complication rates with the intramedullary implant, such as femoral shaft fractures [10-13]. Consequently, modifications of the Gamma**™** nail have been made in recent decades. The diameter of the nail and the valgus ankle were reduced, the lag screw and the targeting device were modified, and a dynamic distal locking option was added, constituting the main changes in the development to the actual Gamma 3**™** nail.

Meanwhile, internationally, many authors have recommended the treatment of unstable pertrochanteric fractures with modern intramedullary implants because of their higher loading capacity and the broad scope of their potential application [14-17]. Evidence exists that intertrochanteric fractures (A3 fractures, according to the AO/OTA classification) of the proximal femur are best treated with intramedullary devices. The best treatment is unclear of A2 fractures with dislocation of the lesser trochanter. Some authors still prefer the sliding hip screw for osteosynthesis [18].

Over the past few years, the Gamma3**™** nail was established in our department for two reasons. Firstly, it can be used for various fractures, e.g., subtrochanteric fractures. Secondly, the Gamma3**™** nail offers standardised and easy handling of the instrumentarium. This ease of use is especially useful in a teaching hospital, in which less experienced residents in training might already utilise it.

To date, there have been few data available on the use of the Gamma3**™** nail in geriatric trochanteric and in subtrochanteric fractures in particular. The aim of this study was to report our experiences with the implementation of the Gamma3**™** nail and our patients’ outcomes in a prospective clinical trial. The actual health care situation should be illustrated. Therefore, few exclusion criteria were applied. In contrast to other trials in particular, multimorbid patients and patients with dementia were included.

## Methods

In our study, patients at least 60 years old were included in this prospective, single-centre, observational study with pertrochanteric and subtrochanteric femoral fractures (ICD 10 S 72.1-72.2) [19].

The criteria for exclusion were polytrauma (ISS≥16) and fractures caused by malignancies or metastases. All of the patients were surgically treated with the Gamma3™ nail (Stryker, Schönkirchen, Germany). The inclusion period was from 1 April, 2009, to 30 September, 2010. Two follow-up visits were performed at 6 and 12 months after trauma, so the observation period ended on 30 September, 2011. Approval of the Ethics Committee of the University of Marburg was obtained (AZ 175/08). Each of the patients gave his or her written consent.

Next, personal data (sex, age) were collected, as well as ASA score, fracture side, and fracture type. The fractures were differentiated according to the AO/OTA classifications for pertrochanteric and subtrochanteric fractures [20].

Data concerning the interval between admission and surgery, the operating time (cutting to suture time), and the operating surgeon were also collected. The surgeons were divided into residents in training and consultants.

Local complications that required surgical intervention were also registered during the hospitalisation period and the follow-up. In addition, systemic perioperative complications, such as cardiac infarctions, strokes, thromboses, and embolisms, were recorded. In-hospital mortality and mortality in the follow-up period were also noted.

The patients were requested to provide information about their function and health-related quality of life retrospectively. The measurements of functional status were the Barthel Index (BI), according to the *Hamburg Classification Manual*[21], and the Instrumental Activities of Daily Living (IADL) [22]. Concerning quality of life, the EQ-5D Index was calculated using the additive lean model, which was described by Greiner et al. [23]. The Barthel Index, the IADL, and the EQ-5D were administered at discharge and during follow-up.

The data were collected into a Filemaker® (FileMaker Inc., Santa Clara, CA, USA) database. Double entry with a plausibility check was performed to improve the data quality.

Predictive Analysis SoftWare (PASW®), version 18.0 (SPSS Inc., Chicago, IL, USA), was used for the data analysis. Descriptive statistics and explorative data analysis were performed. The frequencies for dichotomous variables and the means, standard deviations, and confidence intervals were determined for continuous variables. The patients were divided into different groups: patients without complications, patients with complications, deceased patients, and patients who were operated on either by a resident or by a consultant. Differences in length of hospital stay and duration of surgery between patients who were operated on by residents and by consultants were determined by the Mann–Whitney-U test. The chi-square test was performed to determine differences in the occurrence of local and general complications and mortality. The various results of the Barthel Index, the IADL, and the EQ-5D, prefracture and at the follow-up visits, were compared with the Mann–Whitney-U test. For all of the tests, statistical significance was assumed at *p* < .05.

### Surgical technique and perioperative treatment

Osteosynthesis was usually performed under general anaesthesia. The patients were positioned supine on a traction table. Initially, a closed reduction by axial traction was performed under fluoroscopy. Then, osteosynthesis was performed as previously described [24]. For trochanteric fractures, the Gamma3**™** trochanteric nail (Stryker, Schönkirchen, Germany), with a diameter of 11 mm, a length of 180 mm, and a lag screw angle of 125° or 130°, was used. The long Gamma3**™** nail (360 mm–440 mm) was used for subtrochanteric fractures. If necessary, an open reduction was performed, and a cable wire was used before the nail was inserted. The perioperative treatment of the patients did not vary through participation in this study. All of the patients were treated according to our in-house standards. This process included each of the patients being monitored postoperatively in our intensive care unit. The patients were mobilised on the first postsurgical day with full weight-bearing.

## Results

Out of 90 patients who were included in the present study, 77% were female, and 23% were male. The average age of all of the patients was 81 years old (95% CI 79–82 years). Concerning physical status, most of the patients were classified in the group with ASA 3, with 72% overall. Eleven percent were classified in ASA 4, and 16% in ASA 2. Only 1% of our patients were healthy or had only mild systemic disease (Table 1).

**Table 1:** Patients characteristics

**Data**	**All patients (n=90)**
Average age (years) (±SD) (95% CI)	81 (±8) (79-82)
Gender	
Females	69 (77%)
Males	21 (23%)
Fracture side	
Right	50 (56%)
Left	40 (44%)
Barthel Index (±SD) (95% C e)	82 (±23) (77-87)
ASA* classification (±SD) (95% C)	2.9 (±0.5) (2.8-3.1)
ASA 1	1 (1%)
ASA 2	14 (16%)
ASA 3	65 (72%)
ASA 4	10 (11%)

*ASA = American Society of Anesthesiologists.

The right-to-left proportion of the fractured side totalled 50:40. In total, we treated 88% trochanteric fractures, with only 12% of the fractures being classified as subtrochanteric fractures. In the trochanteric group, most of the fractures we classified in A1 to AO classification, with 36%. Thirty-three percent were A2 fractures, and 19% were A3 fractures. In the subtrochanteric group, we found 9% A1 and 2% A2 subtrochanteric fractures, according to all of the patients. Only 1 patient had an unstable A3 subtrochanteric fracture (Table 2).

**Table 2:** Fracture type, duration of surgery by qualification of the surgeons

**Data**	**All patients (n=90)**	**Qualification of surgeon**	
		**Consultant (n=52)**	**Resident (n=38)**
Fracture type*			
trochanteric A1	32 (36%)	17 (33%)	15 (39%)
trochanteric A2	30 (33%)	17 (33%)	13 (34%)
trochanteric A3	17 (19%)	9 (17%)	8 (21%)
subtrochanteric A1	8 (9%)	7 (13%)	-
subtrochanteric A2	2 (2%)	1 (2%)	1 (3%)
subtrochanteric A3	1 (1%)	1 (2%)	1 (3%)
Duration of surgery in min (±SD) (95% CI)	53 (±31) (46-60)	54 (±34) (45-64)	52 (±29) (42-61)**
Time of hospitalization in days (±SD) (95% CI)	14.1 (±5.1) (13.0-15.2)	14.3 (±5.7) (12.7-15.9)	13.9 (± 4.3) *** (12.5-15.3)

*according to the AO classification.

**p=0.790.

***p=0.461

In 78% (70 patients) of the cases, surgery was performed within 24 hours after admission. Only 1 patient was operated on after more than 48 hours (53 hours), to improve physical conditions before surgery. In total, 16 surgeons (9 consultants, 7 residents) performed the osteosynthesis procedures. Forty-two percent (n=38) of all of the surgeries were performed by residents, with an average experience of 3 years of residency, under the supervision of consultants. Fifty-seven percent (n=52) of all of the operations were performed by consultants themselves. The characteristics of these 2 groups of patients do not differ substantially. Patients who were operated on by residents were slightly younger and had marginally higher average ASA scores and Barthel indices (Table 3). The relationship of operations in the trochanteric group was relatively balanced between surgery by residents and surgery by consultants, with proportions of 54% and 45%. Unlike in the trochanteric group, only 18% of the subtrochanteric fractures were operated on by residents, while 82% of the procedures were performed by consultants. The duration of surgery for all of the fractures amounted to 53 minutes on average, with a range from 19 to 180 minutes. On average, consultants needed 54 minutes, while residents required 52 minutes for surgery (Table 2).

**Table 3:** Characteristics of different patient groups

**Data**	**Patients**				
	**without complication**	**with complication**	**deceased within 12 month**	**operated by consultant**	**operated by resident**
N	78	12	20	52	38
Age (years)					
mean (±SD)	80 (±8.1)	82 (±6.4)	82 (±7.7)	83 (±7.2)	78 (±7.9)
95% CI	78.5 – 82.3	78.4 – 86.6	78 - 85	81 - 85	75 - 80
Barthel Index					
mean (±SD)	82 (±23)	80 (±19)	75 (±25)	80 (±24)***	84 (±21)***
95% CI	77 - 88	67 - 93	62 - 87	73 - 87	77 - 91
ASA* classification					
mean (±SD)	2.9 (±0.6)	2.9 (±0.3)	3.4 (±0.6)**	2.9 (±0.5)	3.0 (±0.6)
95% CI	2.8 – 3.1	2.7 – 3.1	3.1 - 3.6	2.7 – 3.0	2.8 – 3.2

*ASA = American Society of Anesthesiologists.

** p<0.001 in comparison to survivors.

*** p=0.02.

The time of hospitalisation, on average, was 14.1 days (95% CI 13.0-15.2 days).

During hospitalisation, some general complications occurred after surgery. One patient suffered a myocardial infarction, and 1 case of pneumonia and 2 apoplexies were counted in different patients. One patient suffered a ventricular fibrillation during ICU monitoring and was revived successfully.

During hospitalisation, 4 patients died. Two of them had cardio-pulmonary decompensation, 1 had multiple organ failure, and 1 had a cause of death that was unknown (Table 4).

**Table 4:** Complications and mortality by qualification of surgeon

	**All patients (n=90)**	**Qualification of surgeon**		**p-value**
		**Consultant (n=52)**	**Resident (n=38)**	
General in hospital Complication	5 (5.6%)	3 (5.8%)	2 (5.3%)	0.918
Apoplexy	2	1	1	
Embolism	0	-	-	
Myocardial infarction	1	-	1	
Ventricular fibrillation	1	1	-	
Pneumonia	1	1	-	
Thrombosis	0	-	-	
Mortality				
In hospital	4 (4.4%)	4 (7.7%)	0 (0.0%)	0.080
6 month	16 (17.8%)	9 (17.3%)	7 (18.4%)	0.891
12 month	20 (22.2%)	10 (19.2%)	10 (26.3)	0.425
Local complications	8 (8.9%)	4 (7.7%)	4 (10.5)	0.641
Cutting out	1	-	1	
Hematoma	4	3	1	
Deep Infection	1	1	-	
Periosteosynthetic fracture	1	-	1	
Tractus irritation	1	-	1	

In addition, some local complications occurred without significant differences between the groups of surgeons (Table 4). We counted 4 postoperative haematomas and 1 deep infection. Two of the patients with postoperative haematomas received anticoagulants because of pre-existing atrial fibrillation. A third patient had an idiopathic coagulation disorder. One of the haematomas and the infection required surgical interventions. The haematoma was cured in a single step. Three of the haematomas were monitored and treated conservatively. The deep infection was caused by *Staphylococcus aureus*. Several surgical revisions were necessary. The patient died during one of the revisions due to heart failure. The deep infection, which occurred in a patient with a A1 trochanteric fracture, and 3 of the 4 haematomas appeared after surgery by consultants (Table 4). The initial fractures of patients with haematoma were 3 stable A1 and 1 A2 trochanteric fractures.

Implant failures were found in 3 cases during follow-up. All of them occurred after surgery by residents in training (Table 4). One cutting out (A1 trochanteric fracture), 1 tract irritation (A2 trochanteric fracture), and 1 periosteosynthetic fracture (A2 subtrochanteric fracture) occurred.

**Figure 1:**
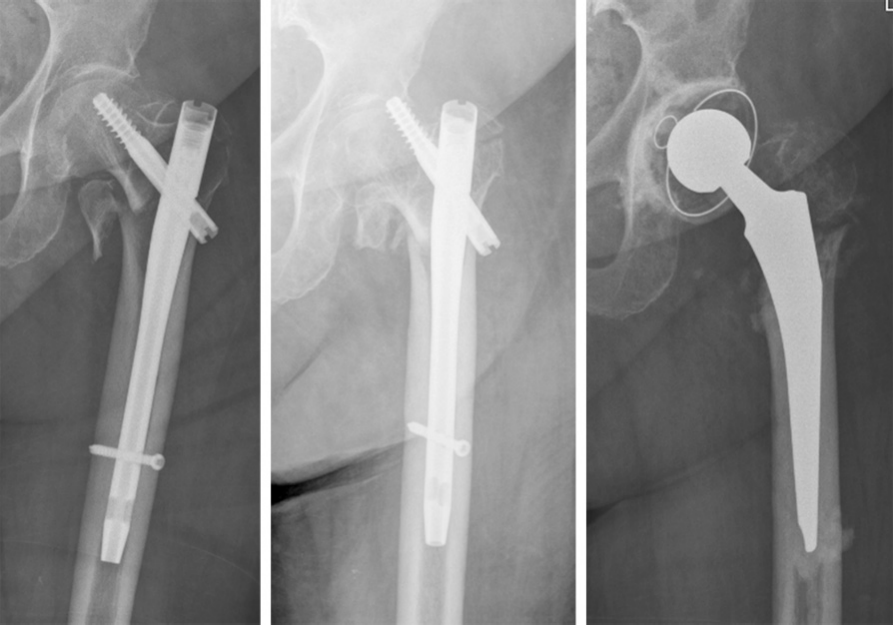
Patient with cutting out: postoperative, during follow-up and after revision with hip prosthesis.

**Figure 2:**
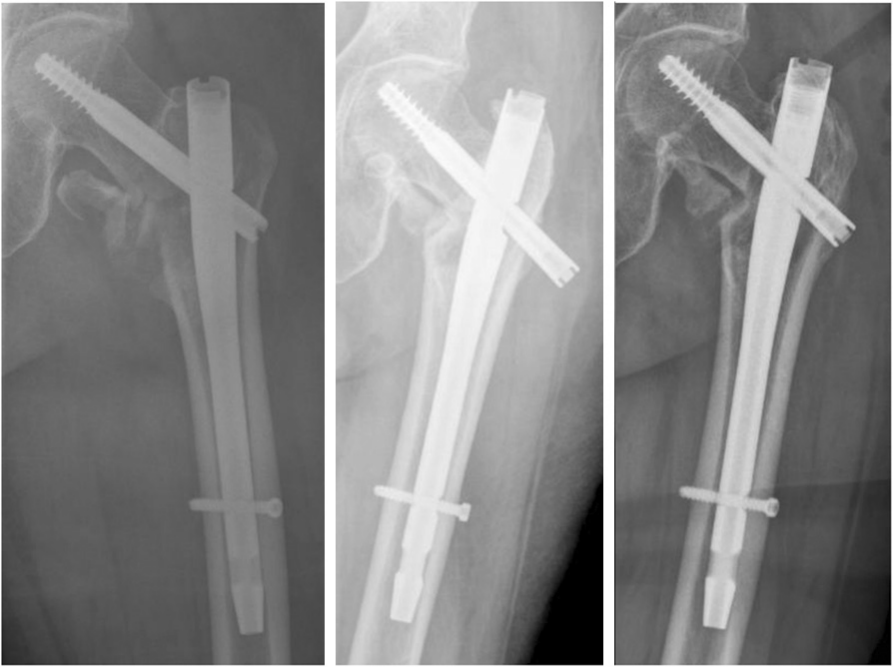
Patient with tract irritation: postoperative, during follow-up and after revision with change of the femoral neck screw.

**Figure 3:**
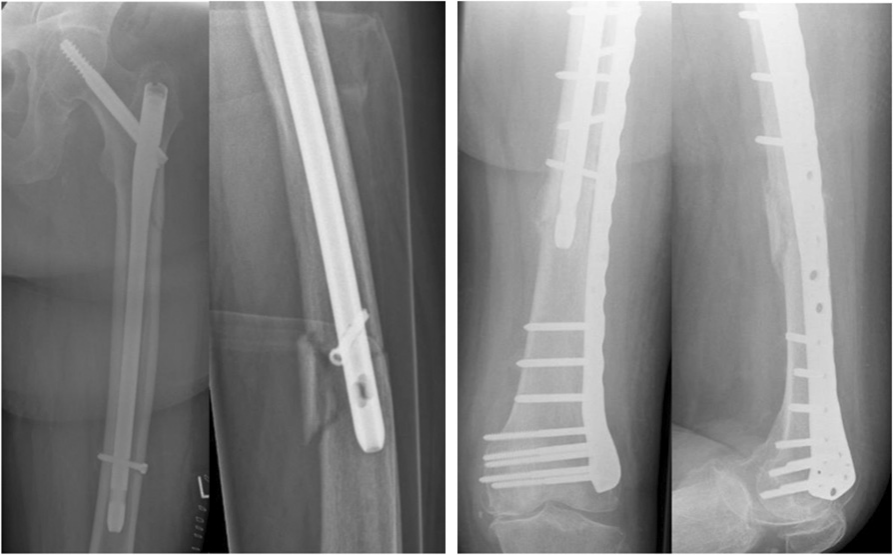
Patient with peri-osteosynthetic fracture: postoperatively, during follow-up and after revision with plate osteosynthesis.

Concerning the cutting out, the nail was removed, and a hip arthroplasty was performed (Figure 1). In the case of tract irritation, only the hip screw was changed without any problems (Figure 2). The patient who suffered the periosteosynthetic fracture was initially treated with a long nail because of a subtrochanteric fracture. The nail was left in, and a plate osteosynthesis was performed without further complications (Figure 3).

Table 3 compares the characteristics of the patients who suffered one of the complications described above, along with the patients who died before the 12-month follow-up. In addition, the patients who neither died nor suffered a complication are described. Patient who were operated on by consultants were significant younger (80 years old, *p* = .02) than patients who were operated on by residents (84 years old). The average ASA score was higher in patients who died within 12 months in comparison to the survivors (*p* < .001). There were no further significant differences between the patients’ characteristics (Table 3).

**Figure 4:**
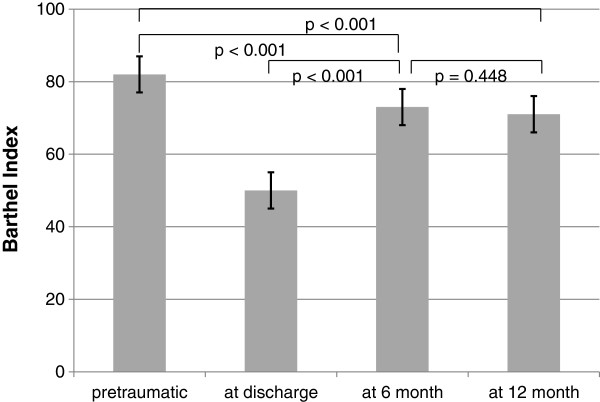
Barthel Index: pretraumatic, at 6 month follow up and at 12 month follow up.

**Figure 5:**
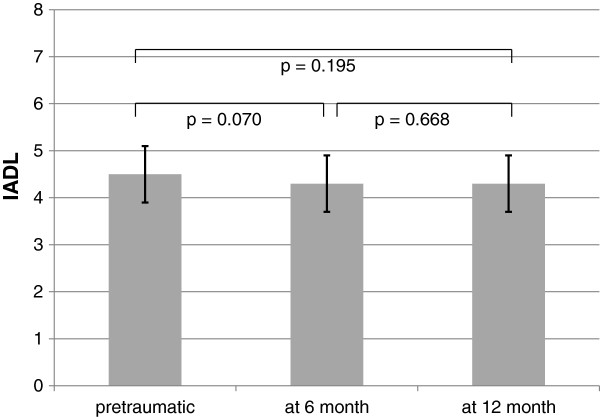
Activities of daily living: pretraumatic, at 6 month follow up and at 12 month follow up.

For the measurement of health status and outcome, several indices were applied. The Barthel Index and IADL were recorded for 11 of the patients with dementia by their relatives. Overall, preoperatively and at hospital release, the Barthel indices of 85 and 86 patients were determined, respectively. The index was 82 (95% CI 77–87) preoperatively and, on average, was lower (50; 95% CI 44–56, *p* < .001) at hospital release (Figure 4). The IADL of the 85 patients preoperatively was 4.5 on average (95% CI 3.9-5.1) (Figure 5).

**Figure 6:**
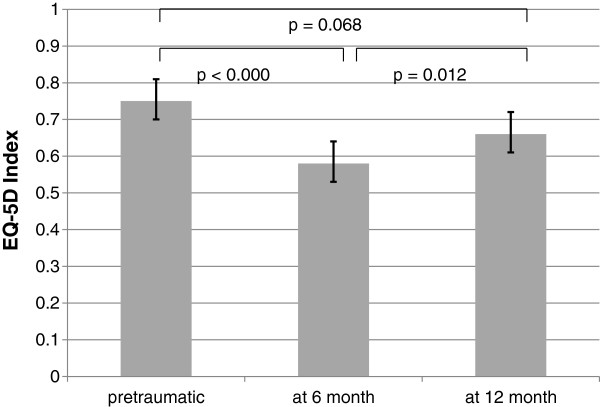
EQ-5D Index: pretraumatic, at 6 month follow up and at 12 month follow up.

The preoperatively determined EQ-5D Index of 74 patients was 0.75 on average (95% CI 0.69-0.80, Figure 6). Sixteen patients could not provide full particulars because of severe dementia.

### Follow-up

All of the patients or their relatives or general practitioners could be contacted and asked about complications or death. Thus, 13 of the 74 patients who were still alive at the 6-month follow-up could not be investigated for the other outcome parameters. At 12 months, 1 more patient was lost to follow-up. The overall follow-up rate was 76 patients out of 90 patients, or 84%.

### Mortality

Twelve patients died during the first 6 months, and another 4 patients died before 1 year was reached. Thus, 12-month mortality totalled 22% without a difference between the surgeons (Table 4).

### Barthel Index and IADL

The Barthel Index increased by approximately 47% (73 index points, 95% CI 66–81) in relation to the status at the hospital release of the patients at 6 months (*p* < .001) but was vaguely similar to the index at follow-up, with 71 index points (95% CI 62–81, *p* = .448), on average, at 12 months (Figure 4). The Instrumental Activities Of Daily Living (IADL) scale amounted to 4.3 on average after 6 months (95% CI 3.4-5.1) and was similar, with 4.3 points on average at 12 months as well (95% CI 3.4-5.3, *p* = .668) (Figure 5).

### Health-related quality of life

The EQ-5D index decreased by approximately 23% (0.58 index points, 95% CI 0.49-0.67, *p* < .001) after 6 months compared to the preoperative status. Thus, in relation to the 6-month follow-up, the EQ-5D index increased by approximately 11% (0.66 index points, 95% CI 0.58-0.75, *p* = .012) after 12 months; however, it undermatched the preoperative status by roughly 11% on average, without reaching a significant difference (*p* = .068, Figure 6).

## Discussion

Trochanteric and subtrochanteric fractures are common fragility fractures in the elderly. While the incidence of proximal femoral fractures is increasing because of demographic transitions [6] in Germany and worldwide [1,3,4], an increase in trochanteric and subtrochanteric fracture incidence can also be predicted. Today, proximal femoral fractures, as typical fragility fractures, are of great socio-economic importance [25].

With its distribution by sex, age, and ASA score (Table 1), our patient collective was typical of fragility fractures of the proximal femur. Studies have shown high polymorbidity in this patient base, with a roughly 50% disposition to ASA classification 3 or 4 [21,26]. The division in this study of different trochanteric and subtrochanteric fracture types also approximately conformed to other studies [27,28]. Thus, our results are comparable to other publications.

To date, the optimal choice for the stabilisation of trochanteric fractures remains controversial because there is still a lack of evidence for the use of intramedullary devices, instead of extramedullary devices [9].

Only for A3 fractures of the proximal femur there is evidence that these fractures are best treated with intramedullary devices. The sliding hip screw is sometimes still preferred for osteosynthesis of A2 fractures [18].

In our department, we have standardised the use of intramedullary devices for the treatment of trochanteric and subtrochanteric fractures. We only implant sliding hip screws for non-displaced femoral neck fractures.

To date, there have been few data available on the use of the Gamma3™ nail in geriatric trochanteric and subtrochanteric fractures in particular. Yaozeng et al. reported good results with the Gamma3™ nail [29], but most of the publications in this context have dealt with the first generation of Gamma™ nail or with other cephalomedullary devices [9]. Improvements of the Gamma3™ nail include a reduction of the diameter of the nail, a change to the valgus angle from 10º to 4º, changes in the design of the femoral neck screw, and the possibility of dynamisation [30].

The aim of this study was to report our experiences with the implantation of the Gamma3™ nail in a prospective, clinical trial. During the observation period, 90 patients who met the inclusion criteria were included in our study.

It could be shown that Gamma3™ nailing was performed by residents in training under the supervision of consults in nearly half of our cases (Table 2). This finding is of great importance in the consideration of further medical education for young physicians, especially in a university hospital setting. One study recently showed that teaching hospitals are $6,000 more expensive in the United States in the operative management of hip fractures. In this context, the 6-month mortality was 1.4% lower than in non-teaching hospitals [31]. In consideration of the duration of surgery and the incidence of complications, such great differences in medical care by residents in training and by consultants appeared less likely in our patient collective (Table 2). The patient characteristics were similar between the patients who underwent surgery by residents and by consultants (Table 3). However, only 2 of 11 subtrochanteric fracture osteosynthesis procedures were performed by residents. This finding might be an indication of the complexity of this type of fracture. In contrast, in the study of Westacott et al., 50% of the surgeries were performed by residents in training [32]. In-hospital mortality (11%) and 1-year mortality (28%) in that study were not affected only by the qualifications of the surgeons. Unfortunately, analyses of further outcome parameters by surgeons were not provided by Westacott et al. [32].

In addition, mechanical failures occurred after surgery by residents in training, bing the great importance of good instruction and careful handling when using the Gamma 3 nail for trochanteric fractures (Table 4). The incidence in our own collective of mechanical failures of 3.3% was lower than the rate reported in actual literature [9-13]. We found 3 implant-related complications during follow-up. Cutting out, tract irritation, and periosteosynthetic fracture occurred only once each. All of these cases required additional surgery but were treated successfully (Figures 1, 2 and 3).

Cutting out is a familiar problem in the osteosynthesis of trochanteric femoral fractures. The meta-analysis of Audige et al. showed an incidence of cutting out in trochanteric fracture surgery of 3.4% with nailing and 1.9% with gliding hip screws [33]. Parker found similar results [9]. In randomised trial, Stern et al. found no difference concerning the cutting out rate when comparing screws to helical blades on either DHS or intramedullary nail [34]. Overall, they found a cutting out rate of 2.2%. Because the tip-apex distance < 25 mm, and the “centre-centre” position of the screw/blade was shown to be advantageous, Stern et al. found 16% of cases with an tip-apex distance > 25 mm and a “centre-centre” position in only 80% of cases [35]. This finding might explain the cutting out rate. We did not perform radiographic examinations in our study, but we found incorrect placement of the femoral neck screw in the only patient with cutting out in the patient sample examined. In the affected patient, the nail was removed, and a hip arthroplasty was performed with a good result.

Another common complication after surgery for trochanteric fractures is iliotibial tract irritation [36]. Only 1 iliotibial tract irritation was seen in our patient sample that required surgery. The femoral neck screw was replaced successfully without any problems.

A periosteosynthetic fracture occurred after the osteosynthesis of 1 of the subtrochanteric fractures and was managed with plate osteosynthesis. According to the authors’ opinion, in the area at the end of the nail, the stress of the femoral shaft was too great. Use of longer nails that extend to the femoral condyle in subtrochanteric fractures could be a solution to this problem.

According to our results, in recent studies, the incidences of perioperative and postoperative femoral fractures after osteosynthesis of trochanteric fractures have decreased, in comparison to older studies. It can be assumed that previous concerns about increased femoral shaft fracture risks with Gamma™ nails have been resolved, with improved implant design and improved learning curves [37].

Our incidence of haematomas seemed to be high (Table 2), but it did not exceed the range in other clinical findings. Wound complications of up to 10% have been described [38]. Only 1 of the haematomas in our collective (1.1%) required surgery and was cured in a single step. Our incidence of deep wound infections was consistent with the literature [9]. In the 1 case of deep wound infection, several surgical revisions were necessary. Unfortunately, the patient died of heart failure during 1 of the revisions. Interestingly, 4 of 5 soft tissue complications appeared after surgery by consultants (Table 4), although 3 of these 4 were stable A1 trochanteric fractures. An explanation for 3 of the 4 postoperative haematomas might have been iatrogenic or idiopathic coagulation disorders.

Fortunately, neither intraoperative complications nor non-unions were recorded. The latter finding can probably be explained by the correct repositioning of the fractures. However, perhaps some non-unions were not detected because X-rays were only performed during follow-up in cases of pain during weight-bearing.

Hospital mortality was 4%, and 1-year mortality was 22% in this study. These rates are low in comparison to recent studies. For example, Barton found a 30-day mortality of 21% and a 1-year mortality of 32% in patients with similar characteristics [39]. The meta-analysis of Liu showed a mortality of 22% [40]. However, in Liu’s analysis, only 2 out of 7 studies had a 1-year follow up. The other 5 studies only had a follow-up period of 6 months or less. In addition, some of the analysed patients were younger than our patients.

Thus, the mortality of our patients was more than double the age-specific mortality rate in Germany. Therefore, the actual mortality rate between 80 and 84 years of age is approximately 5.9% for women and 8.5% for men [41].

The Barthel Index and IADL measurements are internationally accepted assessments for functional physical outcomes in geriatric patients. The Barthel Index was also identified as a strong predictive factor for long-term outcomes after hip fractures [42].

Long-term disability associated with hip fractures has been described in many studies [43]. As expected, the functional status at hospital release tends to decrease. It could be shown that patients improve during follow-up. Certainly, these pretraumatic IADL and Barthel Index values were not reached, as in other studies (Figures 4 and 5) [44].

The Barthel Index fell slightly between the 6- and 12-month follow-ups (Figure 4). According to this finding, even with a 2-year follow-up period, excessive disability, attributable to initial hip fracture, was observed by Magaziner [45]. Overall, the values in our patient collective exceeded the findings of other studies. Kammerlander found a Barthel Index of 49.6 in long-term functional outcomes after hip fractures. However, the patients in that study were older, and the follow-up period was longer (4.9 years) [46]. Thus, over time, further follow-up is planned to obtain more information about the long-term status of the patients.

The EQ-5-D levels were higher at different times of assessment than the levels reported by Ekström et al., although that study only included patients with stable trochanteric fractures. Consequently, the pretraumatic EQ-5D was 0.69, with a value of 0.59 at 12-month follow-up [47]. Concerning femoral neck fractures, a lower quality of life could be shown for displaced, rather than undisplaced, fractures [48]. On the one hand, it can be shown that the EQ-5D values differ between different countries, with higher values for German patients [23]. On the other hand, the trends over the period examined were nearly the same. In this context, it might be interesting to perform more follow-ups later, because Ekström et al. observed an increase of up to 0.66 after 24 months. Interestingly, the Barthel Index fell between the 6- and 12-month follow-ups, while the EQ-5-D Index increased from 0.58 to 0.66 (Figures 4 and 6). It could be that patients become increasingly accustomed to their functional status over time. Furthermore, König et al. showed that even nearly 70% of average citizens aged between 80 and 84 years old report moderate or extreme problems in 1 of the 5 dimensions of the EQ-5-D. Unfortunately, the EQ-5-D indices were not calculated in this article. However, all in all, this finding indicates that health-related quality of life is restricted, even in the normal ageing population, bing the good long-term results of this study.

Mortality, decline in function, and poor quality of life could be attributed in large part to pre-existing conditions. Table 3 shows the characteristics of the different patient groups. Patient who were operated on by consultants were younger and in patients who died within 12 months the average ASA score was higher in in comparison to the survivors. There were no further statistical differences (Table 3). However, some trends indicate that patients who suffer complications are older, are more diseased, and have worse pretraumatic functional statuses. With more patients, including with other types of hip fractures, the predictive value of these parameters could be analysed in detail.

One of the limitations of this study is the retrospective assessment of the Barthel Index, IADL, and EQ-5-D for pre-existing conditions, which allows for bias. Unfortunately, some patients were too senile to provide all of their information. Last but not least, we did not perform a comparison of 2 implants or of surgical procedures, which is why a definitive recommendation of the Gamma 3**™** nail cannot be provided based on this study.

In summary, we successfully illustrated an actual medical care situation concerning the treatment of geriatric trochanteric fractures in our department.

## Conclusion

Surgical treatment of trochanteric fractures with the Gamma3™ nail seems to be a quick and safe procedure, even in vulnerable patient samples. The procedure can also be successfully performed by residents in training, although valuable guidance by consultants seems to be necessary in this kind of surgery. The use of the Gamma3™ could be an improvement in surgery, compared to older cephalomedullary devices. Further studies should be conducted to compare currently available devices for the osteosynthesis of trochanteric fractures.

## Competing interests

The authors declare that they have no competing interests.

## Authors’ contributions

BB, JS and SR made substantial contributions to the conception and design of the present study. The data were collected by BB,TM, JS, CB and DE. BB and TM drafted the manuscript. JS, CB, DE and SR revised the manuscript critically for intellectual content. All of the authors read and approved the final manuscript.
